# Effect of Early Peripheral Parenteral Nutrition Support in an Enhanced Recovery Program for Colorectal Cancer Surgery: A Randomized Open Trial

**DOI:** 10.3390/jcm10163647

**Published:** 2021-08-18

**Authors:** Luis Sánchez-Guillén, Leticia Soriano-Irigaray, Francisco López-Rodríguez-Arias, Xavier Barber, Ana Murcia, M José Alcaide, Verónica Aranaz-Ostáriz, Álvaro Soler-Silva, Andrés Navarro-Ruiz, Antonio Arroyo

**Affiliations:** 1 Colorectal Unit, Department of General Surgery, Elche University Hospital, Miguel Hernandez University, 03202 Elche, Spain; drsanchezguillen@gmail.com (L.S.-G.); franloarias@hotmail.com (F.L.-R.-A.); mjose_alcaide@hotmail.com (M.J.A.); veronica.aranaz@gmail.com (V.A.-O.); soler.medi@gmail.com (Á.S.-S.); arroyocir@hotmail.com (A.A.); 2 Department of Pharmacy, Elche University Hospital-FISABIO, 03203 Elche, Spain; anamurcialopez@gmail.com (A.M.); navarro_and@gva.es (A.N.-R.); 3 Center for Operations Research, Miguel Hernandez University, 03202 Elche, Spain; xbarber@umh.es

**Keywords:** colorectal cancer, postoperative complication, morbidity, peripheral parenteral nutrition, enhanced recovery, oral feeding

## Abstract

Background: Peripheral parenteral nutrition allows repletion of acute nutrient deficiencies and could prevent further nutrition deficits before and after colorectal surgery. A randomized open study was performed to evaluate the effect of perioperative peripheral parenteral nutrition (PPN) support on postoperative morbidity after colorectal cancer surgery within an enhanced recovery program. Methods: Patients were randomized into two groups: peripheral parenteral nutrition (PPN) (with Peri-Olimel N4-E) versus conventional fluid therapy (FT). Ninety-day postoperative complications, laboratory parameters, length of hospital stay, and compliance with the ERAS protocol were assessed. Results: A total of 158 patients were analysed. The overall 90-day complication rate was 38.6% (61 patients), and 24 patients had major complications (Clavien–Dindo III–V) (15.2%). In the multivariate analysis, the intervention (PPN vs. FC) showed a protective effect against postoperative complications (*p* = 0.0031, OR = 0.2 (CI: 0.08–0.87)). Following ordinal regression, PPN and early oral tolerance showed a protective effect, being less likely to develop complications or to move from minor to major complications. In patients with low compliance to ERAS during the first postoperative day, PPN showed a protective effect, preventing 28% of morbidity. Conclusions: Perioperative peripheral parenteral nutrition (PPN) support with Peri-Olimel N4-E in colorectal cancer surgery associated with early oral intake could reduce postoperative complications.

## 1. Introduction

Colorectal cancer (CRC) is still among the most frequently diagnosed cancers, accounting for 1.14 million new cases in 2020, and surgery continues to be the main pillar of treatment [1]. The multimodal enhanced recovery after surgery (ERAS) programs implemented in the last decade have led to substantial improvements in the care of patients undergoing elective colorectal surgery [2]. Designed to reduce perioperative stress, maintain physiological function postoperatively, and promote faster recovery, the widely accepted protocol includes clear recommendations from preoperative to postoperative management. However, postoperative complications remain common and about a third of patients suffer them, with an impact on the length of the hospital stay, costs, and income associated with increased mortality [3,4].

Perioperative nutritional care is one of the pillars of evidence-based ERAS programs, as patients undergoing oncological surgery present an increased risk of malnutrition. Surgical stress and the consequent increase in energy expenditure, weight loss, eating difficulties, and poor appetite decrease nutritional status. Although it is an underestimated value, 10–20% of patients with CRC are malnourished before surgery. Preoperative malnutrition should be corrected or at least improved preoperatively, as it can reduce infectious complications and improve the immune status of the patient [5,6]. Additionally, postoperative nutritional support is crucial in maintaining nutritional status during the catabolic postoperative period, and ERAS protocols support early postoperative feeding within hours after surgery. It has been demonstrated that early oral feeding can improve tissue healing and shorten the postoperative hospital stay, improving clinical outcomes, readmissions, and costs of care [7,8,9,10,11].

However, nutritional therapy during the postoperative recovery period, especially in older patients, is challenging. Decreased appetites, persistent nausea and vomiting, opioid-induced constipation, postoperative ileus, and lack of education about how to optimize their diet lead many patients to not achieving adequate nutritional requirements during the first postoperative days. Because of that, the use of parenteral nutrition (PN) should be considered, as it allows the repletion of acute nutrient deficiencies and prevents further nutrition deficit development and has demonstrated to be safe and effective. Preoperative PN, even 12 h before surgery, has proven to be valuable in stimulating both protein transcription and translation, reducing autophagy and lysosomal degradation, and augmenting the immune system, promoting lymphocyte proliferation in patients undergoing abdominal surgery, and could be beneficial for all patients [12,13,14,15].

Considering the different options for delivering PN, peripheral parenteral nutrition could narrow the nutritional gap in patients before surgery and those recovering after the procedure.

The aim of this study was to evaluate the effect of perioperative peripheral parenteral nutrition (PPN) support in patients undergoing elective CRC surgery versus conventional fluid therapy, improving overall complication rates and shorter stays in the context of an ERAS program.

## 2. Materials and Methods

### 2.1. Study Design and Participants

A single-centre, open, pragmatic, randomized controlled trial was performed comparing the influence of peripheral parenteral nutrition (PPN) (with Peri-Olimel N4-E) versus conventional fluid therapy (FT) on postoperative complications in colorectal surgery patients. Patients with a diagnosis of colorectal cancer between October 2016 and September 2019 treated in a university hospital (designated a Centre of Excellence in ERAS programs) were selected for inclusion. All patients diagnosed with a colorectal tumour scheduled for surgery with preoperative T1-T3NxM0 were included. Patients at severe nutrition risk by one of the ESPEN guidelines criteria (weight loss > 10–15% within 6 months, BMI < 18.5 kg/m^2^, SGA grade C, or NRS > 5, and preoperative serum albumin < 30 g/L (without evidence of liver or kidney dysfunction)) were excluded [8,16]. Additional exclusion criteria were emergency surgery, an American Society of Anaesthesiologists (ASA) physical status IV, renal failure defined as necessitating haemodialysis, hepatic failure, allergy or sensitivity to egg or soy protein, severe bleeding disorder, congenital abnormality of amino acid metabolism, hyperlipidaemia, and inability to comply with the ERAS protocol.

All eligible patients provided written informed consent before undergoing study-related procedures. The study protocol was registered in the NCT register as NCT03606863 and approved by the Ethics Commission of the Elche University Hospital and performed in accordance with the Declaration of Helsinki (World Medical Association, 2013).

### 2.2. Randomization and Masking

Using online randomization software, patients were randomly assigned (1:1) into two parallel groups: the control group (conventional FT) or the experimental group (PPN with Peri-Olimel N4-E). Randomization was done by an external statistician. The investigators, surgeons, patients, and statisticians were unmasked to the group in which the patient was randomly allocated.

### 2.3. Procedures

**Figure 1:**
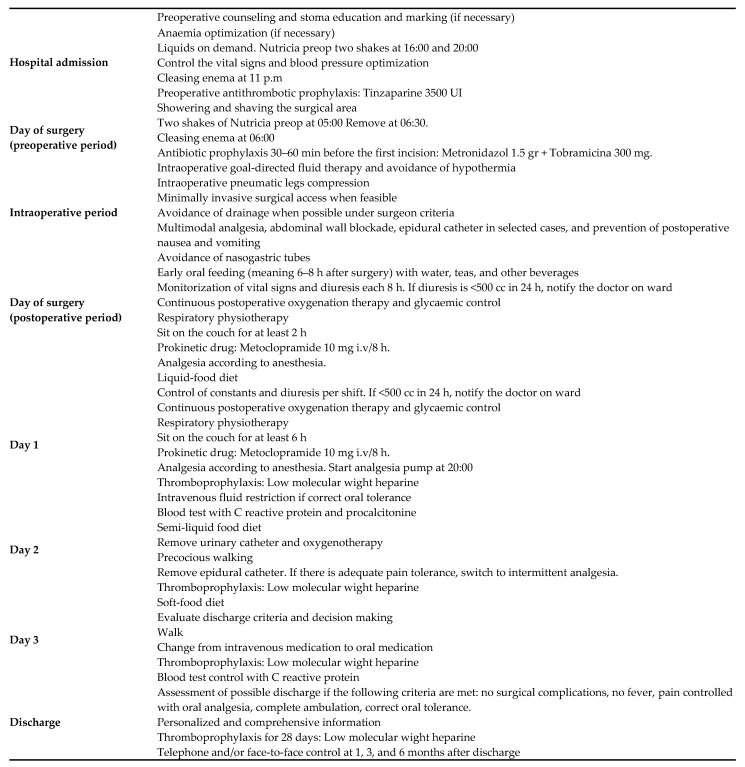
Enhanced recovery program.

All patients were admitted the day before surgery, and patients were preoperatively prepared with only a low fibre diet for three days before surgery. The ERAS bundles used were based on previously published protocols [2]. All the procedures of our ERAS pathway are described in Figure 1. Furthermore, it was required that the patients receive carbohydrate-rich beverages the day before and 2 h before surgery. The control group received conventional FT the day before surgery. The experimental group was treated with peripheral parenteral nutrition (PPN) Peri-Olimel N4E for 4 days (the day before the scheduled surgery and 3 days after surgery). Both groups received antithrombotic therapy and intravenous tobramycin 300 mg and metronidazole 1.5 g at the time of anaesthetic induction. All patients underwent surgery by colorectal surgeons.

#### 2.3.1. Outcome Measures

The primary endpoint was the incidence of postoperative complications, according to the Clavien–Dindo criteria [17]. Minor complications were defined as Clavien–Dindo grades I–II, and major complications were defined as Clavien–Dindo grades III–V. The following variables were analysed as possible risk factors for postoperative complications: demographic data (age, sex), comorbidities (American Society of Anaesthesiologists (ASA) score, oral anticoagulants, smoking habit, high blood pressure, and diabetes), preoperative nutritional status (serum total protein), surgical details (surgical approach, type of anastomosis, perioperative transfusions), and characteristics of the disease (tumour location and TNM stage). Complications and mortality were evaluated at 90 days after surgery using the Clavien–Dindo score. Pathological details were evaluated (TNM system). Analytical (urea, creatinine, haemoglobin, leukocytes, lymphocytes, procalcitonin, and C-reactive protein) and nutritional (serum total protein, albumin, prealbumin, transferrin, and zinc) variables were determined before intervention and daily postsurgery (for the four days after surgery and the day of hospital discharge).

Our ERAS pathway includes a set of interventions from the ERAS protocol (Figure 1). The data on compliance were obtained during the postoperative hospital stay and, in cases of missing data, by a review of patients’ electronic medical charts. Compliance was assessed similarly to Gustafsson et al. [2], including elements before and during the postoperative period. Oral intake and early mobilization were considered crucial interventions for an early diagnosis of postoperative complications during the early postoperative days. Intraoperative ERAS elements and those for whom compliance was nearly 100% were excluded from analysis. A compliance rate ≥ 70% was considered an acceptable level of compliance. Any missing data (written information) about the duration or termination of ERAS interventions were considered noncompliant.

The patients were discharged following the criteria in ERAS, and they were followed for at least 90 days postoperatively. A confidential database was prepared for the collection of data.

#### 2.3.2. Statistical Analysis

The sample size was calculated to compare the incidence of postoperative complications in the control group (patients receiving traditional fluid therapy) versus the intervention group (patients who received early nutritional support with peripheral parenteral nutrition (Peri-Olimel N4-E)). With a confidence level of 95% (alpha = 0.05) and a power of 80% (beta = 0.2) in a bilateral contrast, 170 subjects are required; 85 in the first group and 85 in the second to detect the difference between two proportions as statistically significant, which, for the control group, is expected to be 0.35 and, for the intervention group, is expected to be 0.17, assuming a 10% loss.

Data were collected prospectively, and patients were followed up per protocol with individual case report forms. Continuous variables were reported using the median and interquartile range, while categorical variables were reported using the number of patients and percentage. Differences in the duration of hospitalization between the different groups were analysed with the Kruskal–Wallis test. A univariate analysis was carried out to assess the association between the study variables (major AF, mortality, and morbidity) and the different independent variables: continuous and categorical variables were analysed using the Mann–Whitney U test and χ^2^ tests, respectively. After univariate analysis, we put the variable into a logistic regression model to determine the independent risk factors for a two-level response variable or ordinal logistic regression for categorical ordered response. *p* < 0.05 was considered to indicate statistical significance (two-tailed test). For the multivariate analysis of the response variable, we used two classification methods: (i) logistic regression with a stepwise selection variable method and (ii) regression trees with recursive partitioning for selection variables.

We performed all analyses using R software and the rpart package [18,19].

## 3. Results

### 3.1. Pre- and Perioperative Clinical and Laboratory Features

**Figure 2:**
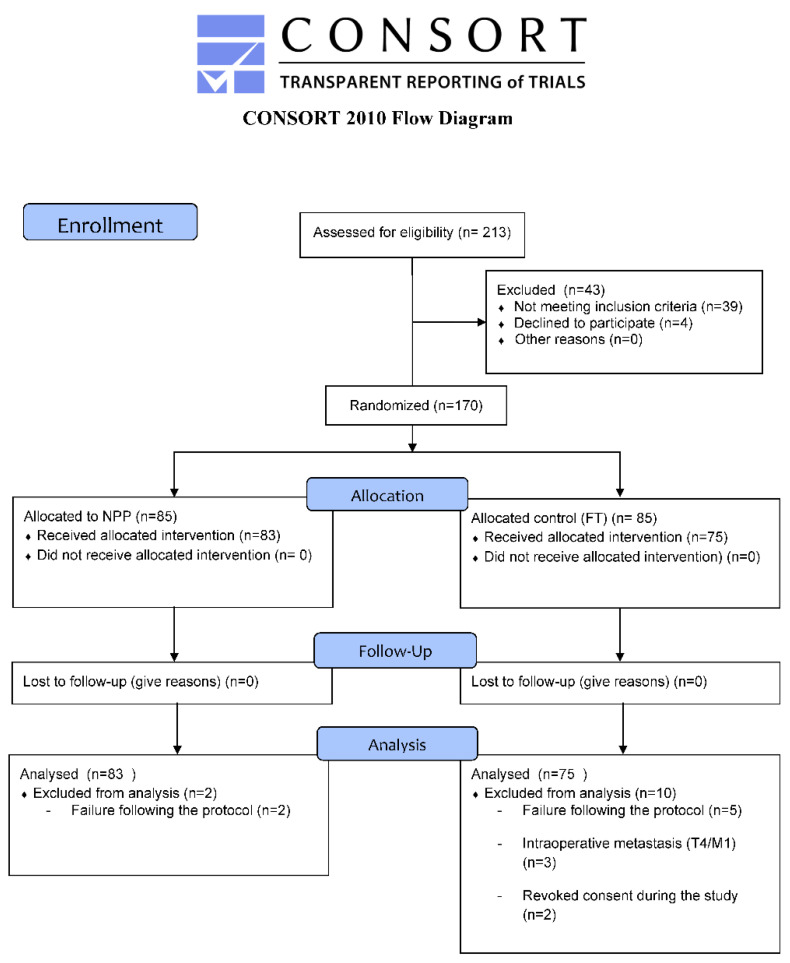
Flowchart of patient in the study.

A total of 170 consecutive patients were allocated for the trial, but 12 were excluded from the analysis according to the pre-established criteria. Figure 2 shows the CONSORT flowchart for the study. Thus, 158 patients were analysed, 83 in the peripheral parenteral nutrition group (PPN) versus 75 patients in the conventional fluid therapy group (FT). Baseline characteristics were similar in both groups. The demographic, preoperative, surgical, and pathological data for the entire sample are detailed in Table 1.

Ninety-seven patients (61.4%) were men and 61 were women, with a median age of 72 years and mainly ASA II (50.6%) and III (40, 5%). All patients underwent scheduled surgery, and the laparoscopic approach was performed in 89.2% of the patients. The most frequent procedures were right hemicolectomy in 54 patients (34.2%) and anterior rectal resection in 44 (27.8%). A stoma was performed in 39 patients (24.6%), of whom 25 underwent a colostomy and 14 underwent an ileostomy. The most frequent type of anastomosis was end-to-end anastomosis in 83 patients (52.5%).

At the time of recruitment, serum total protein, albumin, prealbumin, transferrin, haemoglobin, and zinc and all laboratory parameters were comparable between the two groups, and there were no significant differences between them (Table 2).

### 3.2. Postoperative Changes in Laboratory Parameters

Postoperative changes in laboratory parameters were also comparable between the two groups (Table 2). For both groups, the postoperative serum total protein, albumin, prealbumin, transferrin, haemoglobin, and zinc levels were substantially decreased compared with the preoperative levels. However, there were no significant differences between the groups, and only glucose was higher in the PPN group (118.422 in FT vs. 136.079 in PPN on the first day after surgery (*p* < 0.001), and 96.641 in FT vs. 116.073 in PPN on the third day after surgery (*p* < 0.001)).

### 3.3. Postoperative Complications and Mortality

The median compliance with the measures programmed in the protocol and within the multimodal rehabilitation programs during the preoperative and intraoperative period was 98.6%.

The overall morbidity rate was 38.6% (61 patients), including any deviation in the postoperative course. Thirty-seven patients (23.4%) suffered minor complications (Clavien–Dindo I–II), and 24 patients suffered major complications (Clavien–Dindo III–V) (15.2%). The most frequent complications were anastomosis-related complications (17.7%) followed by surgical site infections (SSIs) (12.6%). Major anastomotic leak was diagnosed in 18 patients (11.4%). The mortality rate was 1.3% (two patients). The median postoperative hospital stay was 6 days (25th–75th percentile: 5–8 days) for the entire group and was lower in the PPN group (6 days (5–8) vs. 7 days (5–9) in the FT group) (*p* = 0.19) (Table 3).

The variables associated with morbidity in the univariate analysis are expressed in Table 4. In the univariate analysis, first day mobilization, first day tolerance for oral feeding, and type of oral feeding on the third postoperative day were related to postoperative morbidity. In the multivariate analysis, the intervention (PPN vs. FC) showed a protective effect against postoperative complications (*p* = 0.0031, OR = 0.2 (CI: 0.08–0.87)), with an 80% lower risk of complications in the group that received PPN.

Following ordinal regression, the risk of postoperative morbidity was established in levels (no complications, minor complications, or major complications). The OR for PPN showed a protective effect, being 73% less likely to develop complications or to move from minor to major complications if the patients received PPN versus the group receiving FT. Additionally, patients with early oral tolerance also were 78% less likely to develop complications or move from minor to major complications (Table 5).

**Figure 3:**
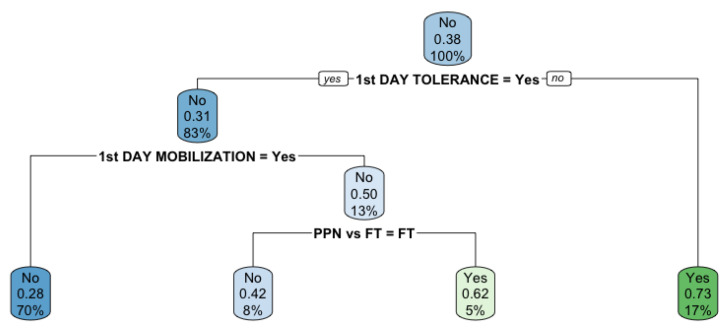
Recursive partition decision tree using Complication “Yes-No” as a response variable with Categorical Variables Related to the Patient. Green color complication and Blue color Non-complication. The left branches represent the subjects who complain about the categorical variable level presented, the right branches represent non-compliance of the categorical level presente in the partition.

Through decision trees, the risk of complications according to the degree of compliance with the ERAS programs during the first postoperative day was established. Patients with no tolerance to oral feeding on the first postoperative day showed a 73% higher risk of postoperative complications. If early postoperative mobilization was not achieved, the risk of postoperative complications increased by 50%. In these cases, with poor compliance during the first postoperative day, PPN showed a protective effect, preventing 28% of postoperative complications (Figure 3).

## 4. Discussion

To the best of our knowledge, this is the first trial that shows that PPN supplementation and early compliance with ERAS programs can reduce postoperative morbidity. Patients receiving PPN had a lower risk of complications than those who received conventional FT, and PPN decreased the chance of worsening complications or developing major postoperative complications by 73%. Compliance with ERAS bundles has a summative impact, decreasing complications, and PPN has shown a protective effect for patients who cannot truly fulfill ERAS protocols because of any deviation in the postoperative course.

The role of peripheral parenteral nutrition in malnourished patients or patients who cannot tolerate oral or enteral nutrition is proven; however, the role of peripheral parenteral nutrition in well-nourished patients who are undergoing colorectal surgery has not yet been investigated. Several recent studies have evaluated the incidence of real malnutrition in well-nourished preoperative patients. Dolan et al. showed that, in patients undergoing CRC surgery, the incidence of sarcopenia is much higher than that described and treated preoperatively and could reach up to 50% [20]. In these patients, intensive perioperative nutrition therapy should be established, especially in fragile and elderly patients [21]. It is thus necessary to improve the nutritional status of these patients with short-term nutritional supplementation. PN provides an adequate and reliable amount of macronutrients and micronutrients, and the intravenous route of administration of nutrients may also allow for rapid improvement in nitrogen balance, increased muscle mass, faster recovery from surgery, improved immune function, and a decrease in the number of general and infectious complications [22]. Low preoperative serum levels of total proteins, albumin, prealbumin, or transferrin have been associated with increased surgical infections, increased morbidity and mortality, and increased hospital stay [23,24,25]. Fasting, reduced protein–calorie intake, and increased catabolic activity triggered by the stress of surgery are reflected in the decrease in analytical parameters such as urea and serum proteins. This decrease seems to be directly related to the degree of surgical stress to which the patient is subjected [26,27]. In our study, we only observed differences in glucose, urea, and zinc postoperative laboratory parameters between the PPN and FT groups in the short-term follow-up. As expected, we did not see differences in total protein and/or serum albumin levels. As serum proteins have a short half-life, they cannot be used to predict changes in the nutritional status of patients in the short term after an intervention, as shown by Rinniella et al. [28]. Peri-Olimel N4-E is a PN emulsion for perfusion by the peripheral route and is composed of amino acids, lipids, and glucose, which can complement enteral and oral nutrition, until the patient has a normal tolerance and is able to reach the minimum daily requirements orally. The osmolarity of the admixture was 750 mOsm/l, which allowed its administration through a peripheral vein. Appropriate osmolarity reduces the risk of peripheral venous thrombophlebitis [29]. It also includes electrolytes (sodium, potassium, calcium, magnesium, phosphate) and can be added to a mixture of trace elements and vitamins. The lipid emulsion is based on olive oil, having a high oleic acid content, which could better preserve the immune response of the patient, decrease oxidative stress, and reduce inflammation, thus improving the healing process, the rate of postoperative infection, and the recovery of the patient [30]. Our results showed an important reduction in all complications, including both major and minor complications. These data suggest that early PPN may modulate the immune response to surgery and sustained postoperative immunosuppression and lead to reduced infectious complications. It is generally believed that major surgery is accompanied by increased catabolism and sustained postoperative immunosuppression, which potentially increases the risk for infectious complications, particularly in patients undergoing surgery for cancer [31]. Similarly, Williams et al. recently observed reduced postoperative major and minor complications with early oral nutritional supplementation (infectious complications (*p* < 0.03), pneumonia (*p* < 0.04), ICU admissions (*p* < 0.04), and gastrointestinal complications (*p* < 0.05) [11].

The importance of maintaining caloric–protein intake leading to better compliance with the ERAS protocol bundles was shown by a significant reduction in the risk of complications. In fact, the consumption of 60% or more of the protein needs during the first 3 postoperative days has been associated with a shorter hospital stay [32]. ERAS programs aim to reduce metabolic stress caused by surgical trauma while supporting early recovery of the patient. They are based on the fulfilment of different bundles (a package of four or five measures aimed at preventing an adverse event) at different moments of the operative process with a demonstrated effectiveness [33,34]. The optimization of nutritional status is an integral component of these programs, included in the bundles for the preoperative period (maintaining carbohydrate-loaded intake up to 2 h before surgery), intraoperative period (optimization of the infusion of fluids), and the postoperative period (early restart of oral nutrition). However, other bundles (minimally invasive surgery, early mobilization, removing all drains early, and so on) also have a summative effect when applied synergistically to obtain significantly better results than when implemented in isolation [35]. Early oral nutrition, especially within 24 h of surgery, improved the results of patients undergoing colorectal surgery with a statistically significant reduction in the length of hospital stay and in the risk of total postoperative complications, and should be proposed as the initial route for postoperative nutrition [7,11]. However, some patients deviate from the normal postoperative course and cannot follow ERAS protocols for various reasons. It is in these patients where we find the current challenge, as they cannot be nourished orally during the first postoperative days. In these patients who cannot tolerate oral feeding as established by the ERAS protocols, PPN has been shown to be a good alternative. The present results show how adding parenteral nutritional support during the immediate postoperative period together with oral feeding can reduce the risk of any kind of postoperative complication (minor and major complications). The rate of major complications and the risk of going from minor to major complications is lower in patients with NPP. Therefore, once the patients have been operated on, postoperative nutritional support should be considered, especially when it is difficult to predict postoperative oral intake. In this study, compliance with ERAS protocols during the early postoperative period allowed us to determine the risk of postoperative complications. Through decision trees, we can establish, during the first postoperative day, the risk of complications according to the degree of compliance with the ERAS programs and adding PPN, also decreasing the risk of postoperative morbidity. This could be an important support and a viable decision-making system for increasing patient protection during the early postoperative days. It is also true that some of the most significant concerns about preoperative PN are catheter-related complications and PN-related complications. However, these complications are not common (overall catheter-related infection occurred in 0–10.7% of patients receiving preoperative PN, pneumothorax in 0–6.7% of patients, phlebitis in 0–1.8%, air embolus 0–1.6%, and thrombosis 0–0.5%) and they were relatively easy to detect and to treat. Appropriate catheter care is a measure to prevent infectious complications [36].

To the best of our knowledge, this study represents the largest randomized study analysing the perioperative effect of PPN in colorectal cancer published thus far. Even if limited by the fact of having been performed in a single centre and including all type of colorectal procedures, the study has strengths. Both groups were comparable, and nutritional supplementation was standardized. Performed in a referral hospital with a dedicated colorectal unit with excellence in ERAS programs and a close follow-up of patients, it offers a real picture of the outcome of ERAS compliance. We included all consecutive patients with colorectal cancer who fulfilled the inclusion criteria, thereby removing bias. In addition, risk factors for complications were evaluated, and confounding factors were removed by means of multivariable regression analysis. Clavien–Dindo scores have the advantage of being able to homogenize patients based on the severity of the complications developed. However, some differences about the type of complications are lost. Although this was a randomized study, we cannot identify the type of complications prevented, which allows to identify that the risk of developing some complication as well as major complications (Clavien–Dindo III–V) (those that require a new interventional procedure or that can be life-threatening and, therefore, the most potentially dangerous) is reduced. Further studies will be necessary to determine in which of these complications this therapy is most useful. Furthermore, this study provides decision trees representing a valuable tool for the early decision-making process evaluating ERAS compliance easily, allowing the addition of PPN in patients with a significant reduction in postoperative complications.

## 5. Conclusions

This randomized open trial demonstrates the benefits of proving early perioperative PPN in patients undergoing colorectal surgery. This is the first trial that shows that PPN supplementation and early compliance with ERAS programs can reduce postoperative morbidity. Patients receiving PPN had a lower risk of complications than those who received conventional FT, and PPN decreased the chance of worsening postoperative complications or developing major complications. It also revealed the importance of postoperative compliance with ERAS bundles during the first postoperative days. For patients who cannot truly fulfil ERAS protocols because of any deviation of the postoperative course, PPN has shown a protective effect on postoperative complications, defining a clear pathway that can help in these challenging patients.

## Figures and Tables

**Table 1:** Demographic, preoperative, surgical, and pathological data of study population.

		FT (*N* = 75) (%)	PPN (*N* = 83) (%)	TOTAL (*N* = 158)	*p*-Value
Age (mean SD)		67.8 (11.6)	71.4 (11.0)	69.7(11.4)	0.049
Sex	Male	46 (47.4)	51 (52.6%)	97 (61.4)	1.000
	Female	29 (47.5)	32 (52.5%)	61 (38.6)	
ASA score	1	10 (71.4)	4 (28.6%)	14 (8.9)	0.139
	2	38 (47.5)	42 (52.5%)	80 (50.6)	
	3	27 (42.2)	37 (57.8)	64 (40.5)	
Surgical approach	Open	9 (52.9)	8 (47.1)	17 (10.8)	0.845
	Laparoscopic	66 (46.8)	75 (53.2)	141 (89.2)	
Type of procedure	Abdominoperineal excision	13 (72.3)	5 (27.7)	18 (11.4)	0.551
	Left hemicolectomy	4 (40)	6 (60%)	10 (6.3)	
	Subtotal colectomy	2 (50)	2 (50%)	4 (2.5)	
	Total colectomy	0 (0)	1 (100)	1 (0.6)	
	Hartmann	1 (50)	1 (50)	2 (1.3)	
	Right hemicolectomy	25 (46.3)	29 (53.7)	54 (34.2)	
	ULAR	2 (50)	2 (50)	4 (2.5)	
	Anterior resection	19 (43.18)	25 (56.82)	44 (27.8)	
	Sigmoidectomy	9 (42.85)	12 (57.15)	21 (13.3)	
Stoma	0	53 (44.9)	66 (55.1)	119 (75.4)	0.402
	1	22 (56.5)	17 (43.5)	39 (24.6)	
Type of stoma	Colostomy	16 (64)	9 (36)	25 (15.8)	0.307
	Ileostomy	5 (35.7)	9 (64.3)	14 (8.9)	
Baseline disease	Left colon cancer	6 (42.8)	8 (57.2)	14 (8.9)	0.651
	Right colon cancer	19 (41)	27 (59)	46 (29.1)	
	Transverse colon cancer	4 (66.7)	2 (33.3)	6 (3.8)	
	Rectal cancer	35 (52.2)	32 (47.8)	67 (42.4)	
	Sigmoid colon cancer	11 (44)	14 (56)	25 (15.8)	
Anastomosis configuration	Side to side	25 (48.1)	27 (51.9)	52 (32.9)	0.280
	End to side	0 (0)	3 (100)	3 (1.9)	
	End to end	36 (43.4)	47 (53.6)	83 (52.5)	
	No anastomosis	14 (70)	6 (30)	20 (12.7)	

ULAR: ultra low anterior resection.

**Table 2:** Values of the parameters analyzed before and after surgery.

	Day	FT (*N* = 75)	PPN (*N* = 83)	*p*-Value
GLUCOSE, mean (SD)	Before surgery	106.798 (28.075)	108.406 (25.622)	0.706
	First day after surgery	118.422 (21.159)	136.079 (36.321)	**<0.001**
	Third day after surgery	96.641 (18.586)	116.073 (30.522)	**<0.001**
UREA, mean (SD)	Before surgery	41.199 (17.823)	44.319 (15.473)	0.239
	First day after surgery	31.678 (13.25)	38.243 (15.492)	**0.005**
	Third day after surgery	34.105 (18.152)	37.944 (20.903)	0.220
CREATININE, mean (SD)	Before surgery	0.836 (0.272)	0.846 (0.255)	0.795
	First day after surgery	0.74 (0.256)	0.777 (0.296)	0.404
	Third day after surgery	0.796 (0.336)	0.774 (0.424)	0.729
TOTAL PROTEINS, mean (SD)	Before surgery	7.108 (0.603)	6.944 (0.648)	0.102
	First day after surgery	5.644 (0.589)	5.522 (0.609)	0.203
	Third day after surgery	5.598 (0.694)	5.528 (0.63)	0.508
ALBUMINE, mean (SD)	Before surgery	4.058 (0.404)	3.956 (0.484)	0.153
	First day after surgery	3.145 (0.394)	3.053 (0.433)	0.168
	Third day after surgery	3.039 (0.493)	2.996 (0.441)	0.554
PREALBUMIN, mean (SD)	Before surgery	22.128 (5.728)	21.236 (5.052)	0.298
	First day after surgery	16.535 (3.669)	16.807 (3.965)	0.655
	Third day after surgery	13.998 (3.919)	14.472 (3.606)	0.429
HEMOGLOBIN, mean (SD)	Before surgery	12.748 (1.639)	12.82 (1.949)	0.802
	First day after surgery	11.221 (1.759)	11.414 (1.819)	0.497
	Third day after surgery	11.168 (1.859)	11.203 (1.647)	0.900
TRANSFERRINE, mean (SD)	Before surgery	257.348 (54.15)	271.33 (65.302)	0.146
	First day after surgery	201.16 (42.743)	214.134 (46.386)	0.069
	Third day after surgery	185.008 (43.717)	192.315 (49.159)	0.325
ZINC, mean (SD)	Before surgery	63.94 (16.692)	64.774 (14.079)	0.733
	First day after surgery	43.074 (10.037)	35.422 (14.203)	**<0.001**
	Third day after surgery	59.705 (15.865)	48.058 (16.313)	**<0.001**
WBC, mean (SD)	Before surgery	6.039 (2.322)	6.189 (2.125)	0.673
	First day after surgery	9.22 (2.94)	10.964 (3.692)	**0.001**
	Third day after surgery	7.152 (2.893)	7.498 (2.567)	0.426
% NEUTROPHILS, mean (SD)	Before surgery	64.514 (8.692)	63.887 (9.46)	0.665
	First day after surgery	80.241 (5.627)	81.135 (6.161)	0.342
	Third day after surgery	74.029 (9.402)	72.927 (8.522)	0.439
LYMPHOCYTES, mean (SD)	Before surgery	22.91 (7.928)	23.366 (8.923)	0.734
	First day after surgery	11.634 (4.513)	10.876 (5.381)	0.340
	Third day after surgery	14.857 (7.235)	15.487 (7.135)	0.581
PLATELETS, mean (SD)	Before surgery	230.802 (117.353)	230.108 (72.006)	0.964
	First day after surgery	215.17 (102.572)	212.811 (66.995)	0.863
	Third day after surgery	213.432 (97.272)	203.345 (73.989)	0.461
FIBRINOGEN, mean (SD)	Before surgery	412.79 (120.087)	379.33 (115.917)	0.076
	First day after surgery	445.295 (119.332)	440.461 (114.263)	0.794
	Third day after surgery	642.406 (181.08)	654.873 (203.599)	0.685
RCP, mean (SD)	Before surgery	14.71 (29.005)	11.017 (22.532)	0.369
	First day after surgery	60.982 (37.77)	63.545 (45.955)	0.703
	Third day after surgery	114.501 (95.885)	95.086 (67.622)	0.140
PROCALCITONINE, mean (SD)	Before surgery	0.634 (3.664)	0.164 (0.129)	0.245
	First day after surgery	0.545 (1.054)	0.963 (1.687)	0.066
	Third day after surgery	1.631 (3.636)	1.751 (7.424)	0.899

WBC: white blood cells, RCP: reactive C protein.

**Table 3:** Postoperative morbidity details for the whole group of patients.

	FT *N* = 75 (%)	PPN *N* = 83 (%)	*p*-Value
Postoperative complications	33 (0.44)	28 (33.7)	0.186
Major complications (C–D III–V)	14 (18.7)	10 (12)	**0.001**
Minor complications (C–D I–II)	19 (25.3)	18 (21.7)	**0.001**
Anastomotic leak	15 (20)	13(15.6)	0.062
Major leak	12 (16)	6 (7.2)	**0.001**
Minor leak	3 (4)	7 (8.4)	**0.001**
Postoperative ileus	12 (16)	13 (15.7)	0.954
Surgical site infections (SSI)	11 (14.6)	9 (10.8)	0.47
Other complications	7 (9.3)	5 (6)	0.433
Length of hospital stay (LOS)	7 (5–9)	6 (5–8)	0.19

PPN: parenteral peripheral nutrition, FT: fluid therapy, C–D: Clavien–Dindo. Chi-Square was used for determined the association of variables under study (bilateral significance).

**Table 4:** Association of categorical variables related to the patient, the surgery, and the tumor with morbidity at univariate analysis (χ^2^ test) and evaluation of independent risk factors for morbidity by multivariate analysis (logistic regression) with a Wald test for global variable significance.

Variables		Patients without Complications *N* = 97 (61.39) (%)	Patients with Any Complications *N* = 61 (38.61) (%)	*p*-Value ^1^	Odds Ratio ^2^	95 % CI ^2^	*p*-Value ^2^
**Age**	**<65**	36 (37.1)	19 (31.1)	0.7133	1		0.4262
	**65–75**	25 (25.8)	16 (26.2)		0.43	(0.1, 1.64)	
	**>75**	36 (37.1)	26 (42.6)		0.77	(0.19, 3.07)	
**Gender**	**Male**	62 (63.9)	35 (57.4)	0.5129	1		0.4441
	**Female**	35 (36.1)	26 (42.6)		1.49	(0.53, 4.16)	
**ASA score**	**I-ii**	58 (59.8)	36 (59)	0.6188	1		0.736
	**Iii**	39 (40.2)	25 (41)		0.82	(0.25, 2.58)	
**PPN**	**No**	42 (43.3)	33 (54.1)	0.2461	1		**0.0031**
	**Yes**	55 (56.7)	28 (45.9)		0.2	(0.06, 0.59)	
**Surgical approach**	**Open**	7 (7.2)	10 (16.4)	0.1215	1		0.7451
	**Laparoscopy**	90 (92.8)	51 (83.6)		0.76	(0.15, 3.9)	
**Stoma**	**No**	79 (81.5)	40 (65.6)	0.016	1.47	(0.57, 5.26)	0.5412
	**Yes**	18 (18.5)	21 (34.4)		1		
**1st day movilization**	**No**	16 (16.5)	25 (41)	<0.001	1.39	(0.3, 7.37)	0.678
	**Yes**	81 (83.5)	36 (59)		1		
**1st day tolerance**	**No**	7 (7.2)	20 (32.8)	<0.001	1		0.0950
	**Yes**	90 (92.8)	41 (67.2)		0.24	(0.04, 1.28)	
**2nd day diet**	Clear liquid/ Full liquid diet	72 (74.2)	42 (68.9)	0.5813	1.7	(0.57, 5.1)	
	Pureed food/ soft food diet	25 (25.8)	19 (31.1)		1		0.34044
**3rd day diet**	Clear liquid/ full liquid diet	24 (24.7)	25 (41)	0.0486	1		0.8575
	Pureed food/ soft food diet	73 (75.3)	36 (59)		0.9	(0.3, 2.83)	

^1^ univarate analysis; ^2^ multivariate analysis. ASA: American Sociecity of Anesthesiology, PPN: peripheral parenteral nutrition.

**Table 5:** Multivariate ordinal logistic regression for complication response variable taking account the order for non-complication vs. minor vs. major complications. Proportional odds ratios are shown.

Variables		OR (95% CI)
**Age**	**<65**	
	**65–75**	0.51 (0.13–1.8)
	**>75**	1.15 (0.32–4.11)
**Gender**	**Male**	1
	**Female**	1.92 (0.75–4.92
**ASA score**	**I-II**	1
	**III**	0.63 (0.20–1.86)
**PPN**	**No**	1
	**YES**	**0.27 (0.09–0.72)**
**Surgical** **approach**	**Open**	1
	**Laparoscopy**	0.66 (0.17–2.57)
**1st day** **mobilization**	**No**	1
	**Yes**	1.14 (0.29–4.82)
**1st day** **tolerance**	**No**	1
	**Yes**	0.22 (0.05–0.98)
**3rd day** **diet**	**Clear liquid/** **Full liquid diet**	1
	**Pureed food/** **Soft food diet**	1.17 (0.43–3.38)

OR: odds ratio, ASA: American Society of Anesthesiology, PPN: peripheral parenteral nutrition.

## Data Availability

The data presented in this study are available on request from the corresponding author. The data are not publicly available dure to privacy restrictions.
